# Surgical Delusions and Follies

**Published:** 1885-09

**Authors:** 


*Surgical Delusions and Follies.* By JOHN B. ROBERTS. Pp. 55. Philadelphia: BLAKISTON & CO. 1884.

There is a great deal of sound practical sense in this little volume. How much of surgical practice is mere habit or fashion, and how little of it is based on scientific truth, one may partially infer from the pithy and outspoken remarks of Dr. Roberts. Though in some minor matters we cannot subscribe to the writer's creeds, we are in the main in thorough accord with his statements and beliefs. He lays about him right and left with a heartiness and vigour that are quite refreshing, and even stimulating; attacking many honoured observances, and demolishing many cherished idols. We should like to see a copy in the hands of every young surgeon (by the way, we should like it to be a better-bound copy than this one, which fell to pieces in our hands when we opened it), for he is likely to profit by it: old surgeons, firmly set in their evil ways, would only be aggravated by it, and probably consign it to the ignominious oblivion of the waste-paper basket.

